# Red Rice Bran Extract Alleviates High-Fat Diet-Induced Non-Alcoholic Fatty Liver Disease and Dyslipidemia in Mice

**DOI:** 10.3390/nu15010246

**Published:** 2023-01-03

**Authors:** Narongsuk Munkong, Surasawadee Somnuk, Nattanida Jantarach, Kingkarnonk Ruxsanawet, Piyawan Nuntaboon, Vaiphot Kanjoo, Bhornprom Yoysungnoen

**Affiliations:** 1 Department of Pathology, School of Medicine, University of Phayao, Phayao 56000, Thailand; 2 Division of Nutrition for Health and Sport, Department of Sport and Health Sciences, Faculty of Sport Science, Kasetsart University, Nakhon Pathom 73140, Thailand; 3 Applied Thai Traditional Medicine Program, School of Public Health, University of Phayao, Phayao 56000, Thailand; 4 Division of Biochemistry, School of Medical Sciences, University of Phayao, Phayao 56000, Thailand; 5 Agriculture Program, School of Agriculture and Natural Resources, University of Phayao, Phayao 56000, Thailand; 6 Division of Physiology, Department of Preclinical Science, Faculty of Medicine, Thammasat University, Pathum Thani 12120, Thailand

**Keywords:** red rice bran, non-alcoholic fatty liver disease, dyslipidemia, obesity, high-fat diet

## Abstract

Red rice bran extract (RRBE) is rich in phytonutrients and has been shown to have anti-diabetic, anti-inflammatory, and antioxidant properties. However, its anti-hepatic steatosis and anti-dyslipidemic properties have not been thoroughly investigated. This study examined the aforementioned properties of RRBE, the underlying mechanism by which it alleviated non-alcoholic fatty liver disease in high-fat diet (HFD)-fed mice, and its major bioactive constituents. The mice were divided into four groups based on their diet: (1) low-fat diet (LFD), (2) LFD with high-dose RRBE (1 g/kg/day), (3) HFD, and (4) HFD with three different doses of RRBE (0.25, 0.5, and 1 g/kg/day). The administration of RRBE, especially at medium and high doses, significantly mitigated HFD-induced hepatosteatosis and concomitantly improved the serum lipid profile. Further, RRBE modified the level of expression of lipid metabolism-related genes (adipose triglyceride lipase (ATGL), cluster of differentiation 36 (CD36), lipoprotein lipase (LPL), liver X receptor alpha (LXRα), sterol regulatory element-binding protein-1c (SREBP-1c), SREBP-2, 3-hydroxy-3-methylglutaryl-CoA reductase (HMGCR), and carnitine palmitoyltransferase 1A (CPT1A)) in hepatic or adipose tissues and improved the expression of hepatic high-density lipoprotein cholesterol (HDL-C) cmetabolism-related genes (hepatic lipase (HL) and apolipoprotein A-ǀ (ApoA-ǀ)). RRBE also attenuated markers of liver injury, inflammation, and oxidative stress, accompanied by a modulated expression of inflammatory (nuclear factor-kappa B (NF-κB) and inducible nitric oxide synthase (iNOS)), pro-oxidant (p47^phox^), and apoptotic (B-cell lymphoma protein 2 (Bcl-2)-associated X and Bcl-2) genes in the liver. High-performance liquid chromatography analyses indicated the presence of protocatechuic acid, γ-oryzanol, vitamin E, and coenzyme Q10 in RRBE. Our data indicate that RRBE alleviates HFD-induced hepatosteatosis, dyslipidemia, and their pathologic complications in part by regulating the expression of key genes involved in lipid metabolism, inflammation, oxidative stress, and apoptosis.

## 1. Introduction

Obesity and its associated cardiometabolic risk factors have become an epidemic worldwide [1]. In parallel, non-alcoholic fatty liver disease (NAFLD) is a common hepatic feature of obesity-related metabolic dysfunction, with an estimated worldwide prevalence of 32.4% [2]. Non-alcoholic fatty liver disease is characterized by the excessive storage of lipids in droplet form within the hepatocytes (simple hepatic steatosis) and may progress to more advanced stages and complications, such as non-alcoholic steatohepatitis (NASH), liver injury, and dyslipidemia [3]. Mechanistically, lipases, such as adipose triglyceride lipase (ATGL) and hormone-sensitive lipase (HSL) hydrolyze triglycerides (TG) within insulin-resistant adipocytes release free fatty acids (FFA) into the circulation, which leads to an increased hepatic FFA uptake through the FA transporters, such as a cluster of differentiation 36 (CD36), and the consequent conversion to TG [4,5]. Meanwhile, de novo lipogenesis and cholesterogenesis lead to hepatic lipid accumulation and hyperlipidemia, primarily through the activation of lipogenic transcription factors called liver X receptor (LXR) and sterol regulatory element-binding protein-1c (SREBP-1c), and cholesterogenic regulators SREBP-2 and 3-hydroxy-3-methylglutaryl-CoA reductase (HMGCR), respectively [4,6]. The impairment of a rate-limiting FA oxidation enzyme, carnitine palmitoyltransferase 1 (CPT1), and a rate-limiting TG hydrolysis enzyme, lipoprotein lipase (LPL), is also important mechanism in the development of hepatic lipid accumulation and dyslipidemia [6,7]. In addition to hyperlipidemia, low high-density lipoprotein cholesterol (HDL-C) levels are observed in metabolic syndrome and NAFLD, which could result from the combination of increased HDL-C clearance due to the activation of hepatic lipase (HL) and the impaired synthesis of HDL-C components such as apolipoprotein A-ǀ (ApoA-ǀ) [8]. The numerous hepatic complications of NAFLD/NASH are associated with the hepatic accumulation of lipotoxic metabolites and dyslipidemia, including inflammation, oxidative stress, and apoptosis, which are the major pathogenic mechanisms underlying NAFLD/NASH-related hepatic injury [3,4,9]. These mechanisms may be mediated by several pathways and crosstalk between them, such as the activation of nuclear factor-kappa B (NF-κB)-mediated inflammatory responses, reactive oxygen species (ROS)-generating nicotinamide adenine dinucleotide phosphate oxidase (NOX), and B-cell lymphoma protein 2 (Bcl-2)-associated X (Bax)-dependent apoptosis [4,9]. Therefore, interventions that prevent the development of metabolic dysfunction, inflammation, oxidative stress, and apoptosis in the liver and other central metabolic organs could be important strategies for attenuating NAFLD, dyslipidemia, and their associated complications, as well as concomitant hepatic injury.

Pigmented or colored rice with its bran is known to contain high contents of nutrients and phytochemicals that have potential health benefits, such as the ability to prevent chronic non-communicable diseases [10]. Rice bran, especially red and purple/black rice bran, reportedly has beneficial properties, such as metabolic-improving, anti-inflammatory, and antioxidant properties, possibly due to the presence of phenolic compounds such as proanthocyanidins, anthocyanins, protocatechuic acids, γ-oryzanols, vitamin E, and coenzyme Q10 [10,11,12,13,14]. We reported in our previous study that red rice bran extract (RRBE) improves glucose-insulin homeostasis by up-regulating insulin-signaling gene expression in the hepatic, adipose, and muscle tissues of obese mice [15]. Moreover, RRBE, along with phenolic compounds, flavonoids, and their subclasses, has inhibitory effects on the production of inflammatory markers and genes in the adipose tissues of obese mice and inhibits macrophages, likely by affecting the expression of NF-κB pathway genes and stimulators of the interferon gene (STING) signaling pathway, respectively [11,12]. Also, other rice varieties such as purple rice and brown rice bran have been reported to exhibit anti-dyslipidemic and antioxidant properties in HFD-fed animals [16,17]. Compared with purple-pigmented and non-pigmented rice bran extracts, RRBE shows the highest content of phenolic compounds, flavonoids, and proanthocyanidins concomitant with its high free radical-scavenging capacity, as observed using in vitro models [13]. Based on these previous reports, we infer that RRBE may be useful in the prevention and treatment of NAFLD and dyslipidemia. However, the molecular mechanisms underlying its anti-NAFLD and anti-dyslipidemic actions are still unknown. Thus, the main aim of our study was to determine the effects of RRBE on NAFLD, dyslipidemia, and its hepatic complications, including inflammation, oxidative stress, and apoptosis, in high-fat diet (HFD)-fed mice, a well-known in vivo model for investigating the metabolic regulatory activities and the mechanism of action of the extracts or phytochemicals. Furthermore, we investigated the presence of some bioactive compounds, including protocatechuic acid, γ-oryzanol, vitamin E, and coenzyme Q10, in RRBE.

## 2. Materials and Methods

### 2.1. Preparation of Ethanol Extracts from Red Rice Bran Samples and Determination of Bioactive Compound Constituents

Hawm Dowk Mali Deang, or Red Hawm rice (*Oryza sativa* L.), was purchased from the Mae Chai Agricultural Cooperative Ltd., Phayao, Thailand, and was milled to obtain its bran using a mini-rice milling machine at Phayao Rice Seed Center, Phayao. In brief, rice bran was extracted thrice using 50% ethanol at a ratio of 1:6 for 72 h at room temperature. Subsequently, the rice bran extracts were filtered using Whatman filter paper no. 1. The filtrate was evaporated and freeze-dried. The freeze-dried extract was stored at −20 °C until use. Bioactive compounds in RRBE, including protocatechuic acid, γ-oryzanol, vitamin E, and coenzyme Q10, were quantified using high-performance liquid chromatography analytical methods, as detailed in previous reports [13,18,19], with some modifications. The concentrations of each compound in the sample were calculated using the calibration curve obtained from various concentrations of the reference standard compounds. The results were denoted as μg/g of dry extract.

### 2.2. Animal Experiment

Animal experimentations were approved by the Institutional Animal Care and Use Committee of University of Phayao, Phayao, Thailand (59 01 04 0037). The animals were housed under standard laboratory conditions (temperature: 22–25 °C; 60% humidity; 12/12 h light/dark cycle) and allowed access to food and water ad libitum. Obesity and NAFLD were induced in 4-week-old male Institute of Cancer Research (ICR) mice (weighing 20 g, Nomura Siam International Co., Ltd., Bangkok, Thailand) by the administration of high-fat diet (HFD; 45% kcal from fat, Research Diets, New Brunswick, NJ, USA) for 12 weeks, and the control mice were kept on a low-fat control diet (LFD; 10% kcal from fat, Research Diets) [20,21]. After 1 week of acclimation, 36 mice were randomly divided into two different diet groups: LFD (*n* = 12) and HFD (*n* = 24) groups for 6 weeks to induce obesity. They were further divided into six groups of six mice each and treated orally using a feeding tube for the next 6 consecutive weeks as follows: LFD-fed mice treated with distilled water (L); LFD-fed mice treated with 1 g/kg of RRBE (LR1); HFD-fed mice treated with distilled water (H); HFD-fed mice treated with 0.25 g/kg of RRBE (HR0.25); HFD-fed mice treated with 0.5 g/kg of RRBE (HR0.5); HFD-fed mice treated with 1 g/kg of RRBE (HR1). RRBE was dissolved in distilled water and administered to treated mice via the feeding tube. The body weight and energy consumption were recorded weekly. At the end of the experiment, blood was harvested from the hearts of all animals after overnight fasting. The serum was prepared and stored at −80 °C for biochemical analyses. Animal tissues and organs were weighed, immediately frozen in liquid nitrogen, and stored at −80 °C and/or fixed in 10% formalin for biochemical and histological analyses.

### 2.3. Measurement of Serum and Hepatic Biochemical Parameters

After centrifugation of the blood at 3000 rpm for 10 minutes at 4 °C, levels of FFA, TG, total-cholesterol (total-C), low-density lipoprotein cholesterol (LDL-C), HDL-C, aspartate aminotransferase (AST), and alanine aminotransferase (ALT) in the serum (*n* = 6 mice/group) were determined using enzymatic and colorimetric kits (the FFA Assay Kit, Fujifilm Wako Pure Chemical Corporation, Osaka, Japan, and the other assay kit, Erba Diagnostics Mannheim GmbH, Mannheim, Germany) and analyzed according to the manufacturer’s protocols. For the measurement of total lipids, liver tissue from each mouse was homogenized in isopropanol (5 %, *w*/*v*) and centrifuged at 8000 rpm for 15 min. Levels of TG and total-C in the liver supernatants were estimated using the same commercial kits used for the determination of serum lipids, and their results were expressed as mg/g of tissue [22].

### 2.4. Evaluation of Hepatic Histopathological Changes

Liver tissue sections were stained with hematoxylin and eosin (H and E) and were then captured using an Olympus BX53-P polarizing microscope equipped with an Olympus DP21 digital microscopy camera (Olympus Corporation, Tokyo, Japan). Five microscopic fields (×400) from each section (*n* = 6/group) were randomly selected for analysis. For histopathology studies, each captured image was used to observe the common morphological changes of NAFLD, including the accumulation of lipid droplets and inflammatory cells, and subsequently used to quantify the steatosis score based on the proportion of steatosis area in the liver parenchyma (grade 0: none; grade 1: <33%; grade 2: 34–66%; and grade 3: >66%) and inflammation scores based on the presence of inflammatory foci in the liver parenchyma (grade 0: none; grade 1: 1–2 foci/field; grade 2: 3–4 foci/field; and grade 3: >4 foci/field), as previously described [23].

### 2.5. Measurement of Hepatic Nitric Oxide (NO), ROS, and Malondialdehyde (MDA) Levels

The levels of NO and ROS in supernatant from the liver were determined according to the previous protocols [24], with a few modifications using Griess reagent (sulfanilamide and N-1-naphthylethylenediamine dihydrochloride) and fluorescence dye 2′,7′dichlorodihydrofluorescein diacetate (DCF-DA) (Sigma-Aldrich, Saint Louis, MO, USA), respectively. Meanwhile, the hepatic levels of MDA, the product of lipoperoxidative damage caused by oxidative stress, were determined by the double heating thiobarbituric acid-trichloroacetic acid method, as described earlier [25]. Hepatic protein concentrations were determined by Bradford assay reagent (Bio-Rad Laboratories, Inc., Hercules, CA, USA) and subsequently used to normalize the levels of MDA. The results were presented as nmol/mg of protein.

### 2.6. RNA Extraction, cDNA Synthesis, and Real-Time Polymerase Chain Reaction (PCR)

Total RNA was extracted from tissues using TRIzol reagent (Invitrogen; Thermo Fisher Scientific, Inc., Waltham, MA, USA) and used as a template for complementary DNA (cDNA) synthesis using High-Capacity cDNA Reverse Transcription kit (Applied Biosystems; Thermo Fisher Scientific Inc., Waltham, MA, USA) in accordance with the manufacturer’s recommendations. Real-time PCR was conducted on a StepOnePlus Real-Time PCR System using TaqMan reagents (Applied Biosystems), and its reaction was performed as follows: 2 min at 50 °C, 10 min at 95 °C, 40 cycles of 95 °C for 15 s, and 60 °C for 1 min. The relative expression level of each target gene was calculated from real-time PCR cycle threshold (Ct) values using the 2^−ΔΔCt^ method and normalized to the expression level of an internal control gene, glyceraldehyde 3-phosphate dehydrogenase (GAPDH). The assay IDs for TaqMan probes (Applied Biosystems) are listed in Table S1.

### 2.7. Statistical Analysis

All data are expressed as the means ± standard deviation (SD). One-way analysis of variance (ANOVA) with Tukey’s post-hoc multiple comparison test was used to test the statistically significant differences between the means of experimental groups, using IBM SPSS Statistics 25 (IBM Corp., Armonk, NY, USA). A *p*-value lower than 0.05 was used to indicate statistically significant differences between the group means.

## 3. Results

### 3.1. Bioactive Constituents of RRBE

Our previous phytochemical screening of RRBE displayed the presence of total phenolics and their major subclasses, including flavonoids, proanthocyanidins, and anthocyanins [11]. The current study extends this screening to identify other phenolic (protocatechuic acid) and non-phenolic (γ-oryzanol, vitamin E, and coenzyme Q10) bioactive compounds in RRBE, as presented in Table 1. 

### 3.2. RRBE Mitigated Dyslipidemia in HFD-Fed Obese Mice

**Figure 1:**
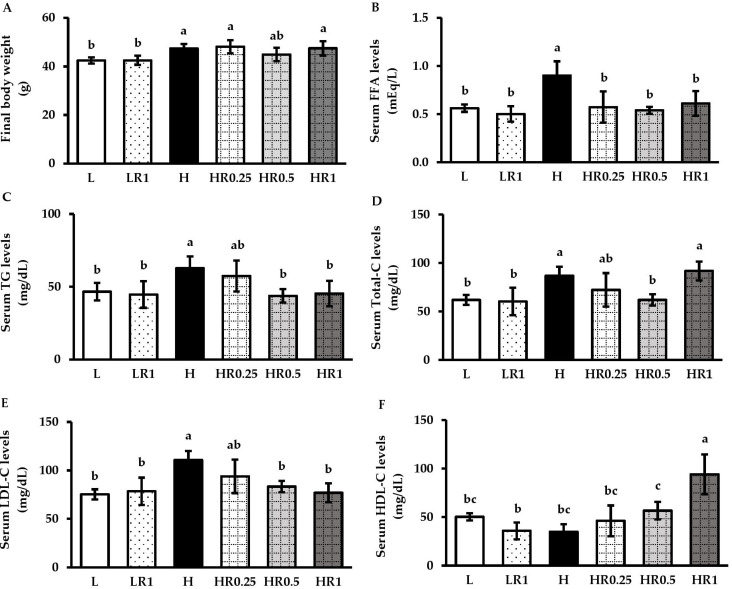
Effects of RRBE on obesity and dyslipidemia in mice fed with experimental diets: (**A**) final body weight; (**B**) serum FFA levels; (**C**) serum TG levels; (**D**) serum total-C levels; (**E**) serum LDL-C levels; (**F**) serum HDL-C levels. Values are means ± SD; 6 mice/group. Statistically significant difference at *p* < 0.05 was analyzed using one-way ANOVA with Tukey’s test and is indicated by different letters above error bars. The L, LFD, and distilled water co-treated group; LR1, LFD, and high-dose (1 g/kg) RRBE co-treated group; H, HFD, and distilled water co-treated group; HR0.25, HFD, and low-dose (0.25 g/kg) RRBE co-treated group; HR0.5, HFD, and middle-dose (0.5 g/kg) RRBE co-treated group; HR1, HFD, and high-dose RRBE co-treated group.

Prior to HFD administration, the initial body weight was not significantly different between the groups (Figure S1A), whereas the body weight of the HFD-fed-only group was significantly higher than those in all the LFD-fed groups after 12 weeks of HFD administration (Figure 1A). The body weights at Week 12 in the RRBE-treated groups were slightly lower than those of the H group. The energy consumption was significantly higher in all the HFD-fed mice compared to the LFD-fed mice, while there were no differences observed between the non-treated and RRBE-treated HFD-fed mice (Figure S1B). In addition, the serum levels of FFA (Figure 1B), TG (Figure 1C), total-C (Figure 1D), and LDL-C (Figure 1E) were significantly higher in untreated HFD-fed mice than in untreated LFD-fed mice after 12 weeks of dietary exposure, while HDL-C levels tended to be lower in HFD-fed mice than LFD-fed mice (Figure 1F). Both FFA and TG in the serum of the HFD-fed mice treated with RRBE, especially at 0.5 and 1 g/kg/day doses, were significantly lower than those in the HFD-fed group without RRBE treatment. Moreover, HFD-fed mice treated with middle-dose (0.5 g/kg/day) RRBE also showed significant mitigations of both total-C and LDL-C levels, but no significant increase in HDL-C levels was observed compared with the H group. In HFD-fed animals treated with high-dose (1 g/kg/day) RRBE, serum LDL-C levels were significantly decreased but serum HDL-C levels significantly increased, with serum total-C levels remaining unchanged. The body weight (Figure 1A), energy intake (Figure S1B), and serum lipid levels (Figure 1B–F) of LFD-fed mice treated with high-dose RRBE were not significantly different from the LFD-fed group without RRBE treatment and HFD-fed groups with RRBE treatments, especially treatments at middle and high doses.

### 3.3. RRBE Alleviated HFD-Induced Hepatosteatosis

**Figure 2:**
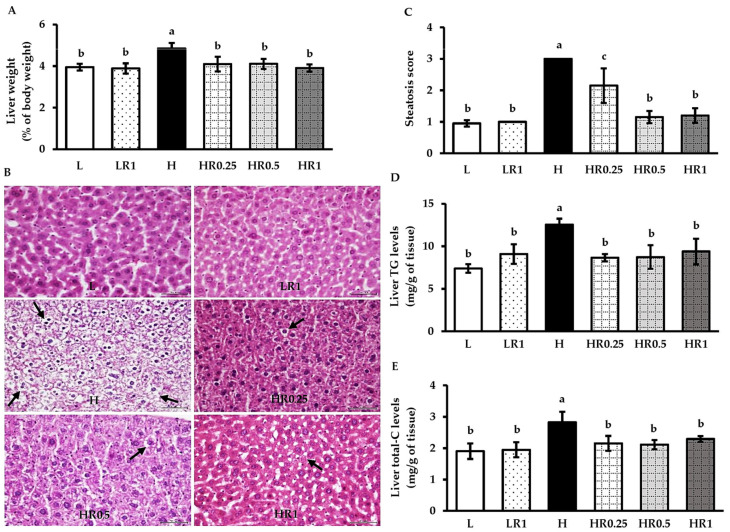
Effects of RRBE on indicators of hepatic steatosis in mice fed with experimental diets: (**A**) liver/body weight ratio; (**B**) representative photomicrographs of H and E-stained liver sections showing the lipid vacuoles (black arrows; ×400 magnification, bar length = 50 µm); (**C**) steatosis score; (**D**) liver TG content; (**E**) liver total-C content. Values are means ± SD; 6 mice/group. Statistically significant difference at *p* < 0.05 was analyzed using one-way ANOVA with Tukey’s test and is indicated by different letters above error bars. The L, LFD, and distilled water co-treated group; LR1, LFD, and high-dose (1 g/kg) RRBE co-treated group; H, HFD, and distilled water co-treated group; HR0.25, HFD, and low-dose (0.25 g/kg) RRBE co-treated group; HR0.5, HFD, and middle-dose (0.5 g/kg) RRBE co-treated group; HR1, HFD, and high-dose RRBE co-treated group.

The ratios of the liver to body weight were significantly increased in the HFD-fed group compared with the LFD control group; however, these values were significantly attenuated in the RRBE-treated HFD-fed groups compared with the HFD-fed group (Figure 2A). Consistent with increased liver weight, the H and E staining of the liver tissue obtained from the H group exhibited hepatosteatosis with a high accumulation of lipid vacuoles in hepatocytes compared with the L group (Figure 2B). The L group displayed a normal arrangement of the hepatocytes, with low intracellular lipid vacuoles separated by sinusoidal space. Using biochemical analyses, we also found that the H group showed significant increases in both hepatic TG (Figure 2D) and total-C levels (Figure 2E). In comparison with the H group, co-treatments of HFD with RRBE in the HR0.25, HR0.5, and HR1 groups markedly alleviated the accumulation of lipid droplets in hepatocytes, as well as decreased TG and total-C contents in hepatic tissue. However, the hepatic lipid vacuoles were higher in RRBE-treated HFD groups than the group fed with LFD alone. Meanwhile, control mice treated with RRBE had hepatic weight, structure, and lipid levels similar to the mice in the control diet group.

### 3.4. RRBE Regulated the Expression of Genes That Encode Key Regulators of Lipid Metabolism

**Figure 3:**
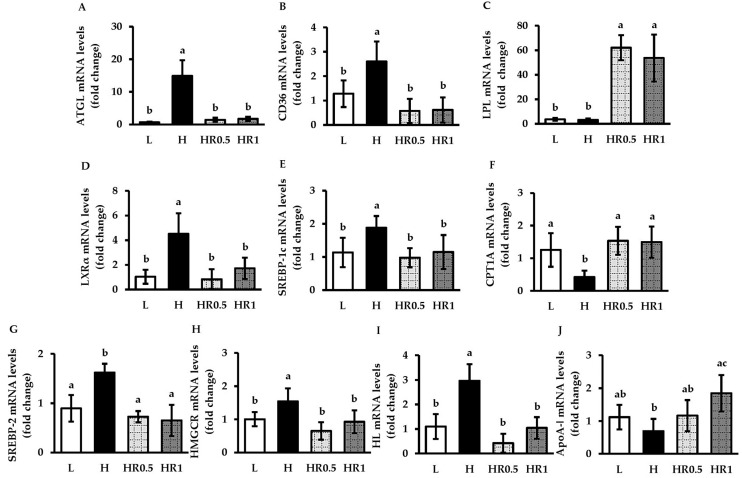
Effects of RRBE on the expression of lipid metabolism-associated genes in mice with HFD-induced NAFLD and dyslipidemia: (**A**) adipose tissue ATGL mRNA levels; (**B**) liver CD36 mRNA levels; (**C**) adipose tissue LPL mRNA levels; (**D**) liver LXRα mRNA levels; (**E**) liver SREBP-1c mRNA levels; (**F**) liver CPT1A mRNA levels; (**G**) liver SREBP-2 mRNA levels; (**H**) liver HMGCR mRNA levels; (**I**) liver HL mRNA levels; (**J**) liver ApoA-ǀ mRNA levels. Values are means ± SD; 6 mice/group. Statistically significant difference at *p* < 0.05 was analyzed using one-way ANOVA with Tukey’s test and is indicated by different letters above error bars. The L, LFD, and distilled water co-treated group; H, HFD, and distilled water co-treated group; HR0.5, HFD, and middle-dose (0.5 g/kg) RRBE co-treated group; HR1, HFD, and high-dose (1 g/kg) RRBE co-treated group.

According to our results shown above in Figure 1 and Figure 2, treatments of HFD-fed mice with RRBE at doses of 0.5 and 1 g/kg/day resulted in the better regulation of dyslipidemia, a better steatosis score, as well as better indicators of inflammation and oxidative stress, when compared to mice treated with RRBE at a dose of 0.25 g/kg/day. Middle and high doses of RRBE were therefore selected to further investigate the molecular mechanisms underlying their metabolic and beneficial effects. Real-time PCR analyses revealed that HFD-fed mice significantly increased mRNA expression levels of ATGL in adipose tissues (Figure 3A), CD36 (Figure 3B), LXRα (Figure 3D), SREBP-1c (Figure 3E), SREBP-2 (Figure 3G), HMGCR (Figure 3H), and HL (Figure 3I) and decreased mRNA expression levels of CPT1A (Figure 3F) in the liver, compared with the LFD-fed mice. The HFD-fed mice also tended to have lower hepatic ApoA-ǀ mRNA levels (Figure 3J) than control diet-fed mice. However, LPL mRNA expression was statistically insignificant in the adipose tissues of HFD-fed mice compared to the controls (Figure 3C). In contrast to the HFD-only group, the consumption of RRBE at doses of 0.5 and 1 g/kg statistically significantly decreased the mRNA levels of ATGL, CD36, LXRα, SREBP-1c, SREBP-2, and HL, but increased LPL and CPT1A mRNA levels in HFD-fed mice. Additionally, liver ApoA-ǀ mRNA levels were significantly increased in HFD-fed mice treated with high doses of RRBE and tended to be increased in HFD-fed mice treated with a middle dose of RRBE.

### 3.5. RRBE Relieved HFD-Induced Hepatic Inflammation

**Figure 4:**
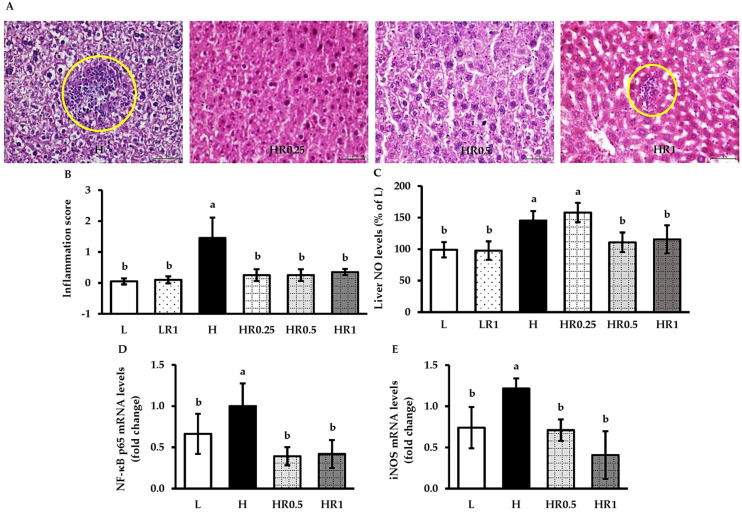
Effects of RRBE on hepatic inflammatory markers in mice fed with experimental diets: (**A**) representative photomicrographs of H and E-stained liver sections showing the presence of inflammatory cell foci (yellow circles; ×400 magnification, bar length = 50 µm); (**B**) inflammatory score; (**C**) liver NO levels; (**D**) liver NF-κB p65 mRNA levels; (**E**) liver iNOS mRNA levels. Values are means ± SD; 6 mice/group. Statistically significant difference at *p* < 0.05 was analyzed using one-way ANOVA with Tukey’s test and is indicated by different letters above error bars. The L, LFD, and distilled water co-treated group; LR1, LFD, and high-dose (1 g/kg) RRBE co-treated group; H, HFD, and distilled water co-treated group; HR0.25, HFD, and low-dose (0.25 g/kg) RRBE co-treated group; HR0.5, HFD, and middle-dose (0.5 g/kg) RRBE co-treated group; HR1, HFD, and high-dose RRBE co-treated group.

According to the hepatic histological evaluations, the presence of inflammatory cell clusters was not evident in any of the LFD-fed groups (Figure 2B). In contrast, the inflammatory cell infiltration and its score were notably exacerbated in the livers of the HFD-fed mice compared with both untreated and RRBE-treated control mice (Figure 2B and Figure 4A,B). RRBE administration alleviated these histopathological changes in the HFD-fed mice. In parallel, the NO levels (Figure 4C) and expression levels of inflammatory genes, namely NF-κB p65 (Figure 4D) and inducible nitric oxide synthase (iNOS) (Figure 4E), in the livers of mice fed with HFD alone were significantly higher compared with the LFD-fed mice, and RRBE co-treatments significantly relieved their levels in the livers of HFD-fed mice.

### 3.6. RRBE Suppressed HFD-Induced Hepatic Oxidative Stress

**Figure 5:**
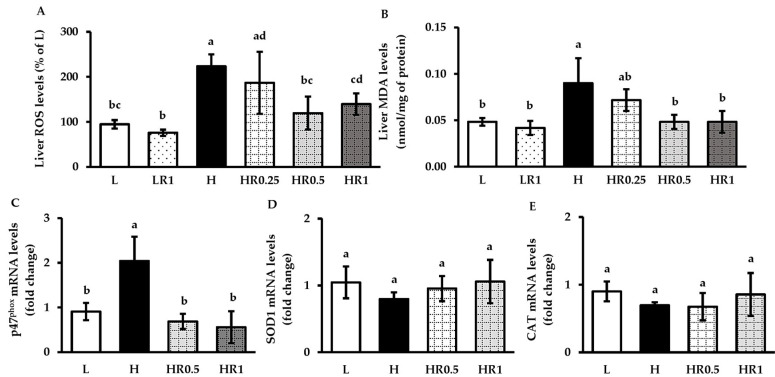
Effects of RRBE on hepatic oxidative stress markers in mice fed with experimental diets: (**A**) liver ROS levels; (**B**) liver MDA levels; (**C**) liver p47^phox^ mRNA levels; (**D**) liver SOD1 mRNA levels; (**E**) liver CAT mRNA levels. Values are means ± SD; 6 mice/group. Statistically significant difference at *p* < 0.05 was analyzed using one-way ANOVA with Tukey’s test and is indicated by different letters above error bars. The L, LFD, and distilled water co-treated group; LR1, LFD, and high-dose (1 g/kg) RRBE co-treated group; H, HFD, and distilled water co-treated group; HR0.25, HFD, and low-dose (0.25 g/kg) RRBE co-treated group; HR0.5, HFD, and middle-dose (0.5 g/kg) RRBE co-treated group; HR1, HFD, and high-dose RRBE co-treated group.

The H group was found to have significantly elevated serum and liver levels of ROS (Figure 5A) and MDA (Figure 5B) compared to the untreated and high-dose RRBE-treated controls. The H group was also found to have significantly up-regulated expression levels of the gene-encoding NOX subunit p47^phox^ (Figure 5C) in the livers of mice. The elevated levels of ROS, MDA, and p47^phox^ mRNA were markedly suppressed in both middle- and high-dose RRBE-treated obese mice compared to non-treated obese mice. However, no significant differences were found in the mRNA levels of both superoxide dismutase 1 (SOD1) (Figure 5D) and catalase (CAT) (Figure 5E) between the experimental groups.

### 3.7. RRBE Reduced HFD-Induced Hepatic Injury and Apoptosis

**Figure 6:**
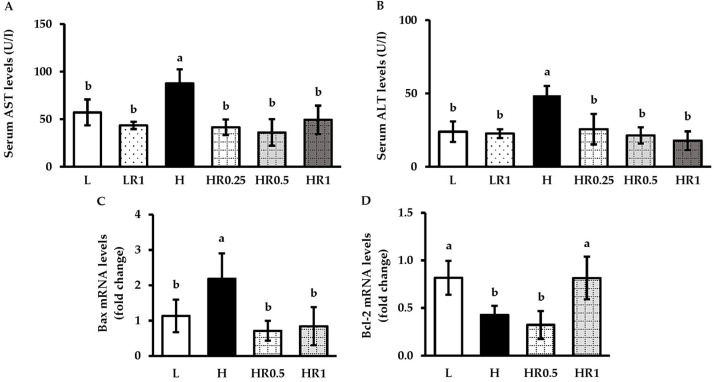
Effects of RRBE on hepatic injury and apoptotic markers in mice fed with experimental diets: (**A**) serum AST levels; (**B**) serum ALT levels; (**C**) liver Bax mRNA levels; (**D**) liver Bcl-2 mRNA levels. Values are means ± SD; 4–6 mice/group. Statistically significant difference at *p* < 0.05 was analyzed using one-way ANOVA with Tukey’s test and is indicated by different letters above error bars. The L, LFD, and distilled water co-treated group; LR1, LFD, and high-dose (1 g/kg) RRBE co-treated group; H, HFD, and distilled water co-treated group; HR0.25, HFD, and low-dose (0.25 g/kg) RRBE co-treated group; HR0.5, HFD, and middle-dose (0.5 g/kg) RRBE co-treated group; HR1, HFD, and high-dose RRBE co-treated group.

As shown in Figure 6A,B, mice in the H group exhibited a significant increase in serum AST and ALT levels compared to mice in both the L and LR1 groups. Consistent with these results, Bax expression levels (Figure 6C) were significantly higher, but Bcl-2 expression levels (Figure 6D) were significantly lower in the livers of the H group than those in the L group. Conversely, significant reductions in serum AST and ALT and hepatic Bax expression were found in mice in both the HR0.5 and HR1 groups compared with those in the H group. The HR1 group also showed significant up-regulation in Bcl-2 mRNA levels compared to the H group, which was absent in the HR0.5 group.

## 4. Discussion

Here, we first characterized the mouse model of HFD-induced dyslipidemia and NAFLD used in our study. Our results showed that high-energy density HFD feeding was able to induce significant serum and hepatic lipid elevations with a significant increase in body weight in mice, which were accompanied by the altered expression of the key genes involved in FFA, TG, and cholesterol metabolism in hepatic and adipose tissues compared to the controls. In parallel with these lipid metabolic dysfunctions, HFD feeding also induced hepatic injury associated with the elevation of inflammatory, oxidative stress, and apoptotic markers in mice. This study indicates that dyslipidemia, NAFLD, and obesity were successfully induced in our model by feeding the mice with HFD, which may be due to the significant increase in their energy intake. Our results are in accordance with those reported by previous studies [20,21,26,27,28,29,30,31]. Thus, this model is appropriate for examining the precise mechanisms underlying the anti-dyslipidemic and anti-NAFLD activities of RRBE. Overall, we found that the treatment of HFD-fed mice with RRBE, containing protocatechuic acid, γ-oryzanol, vitamin E, and coenzyme Q10, resulted in a significant reduction in dyslipidemia, NAFLD, and hepatic damage, which were likely mediated through the regulation of genes related to lipid metabolism, inflammation, oxidative stress, and apoptosis. In addition to the mechanistic study, the results of our study showed that the body weight, liver weight, energy intake, and liver histology, as well as the levels of biochemical, inflammatory, and oxidative stress parameters, were normal in the serum and/or liver of control mice fed with RRBE at a dose of 1 g/kg for 6 weeks relative to the untreated control mice, suggesting RRBE is not toxic. However, further studies are needed to confirm these findings in appropriate models, such as the acute and chronic toxicity studies in rodent models.

Next, we examined the anti-hyperlipidemic and anti-hepatic steatosis activities of RRBE in the HFD-fed mouse model. In the early stage, adipocytes become insulin-resistant and release free fatty acids (FFA) into the circulation through the sequential stimulation of the lipolytic enzymes ATGL, HSL, and monoglyceride lipase (MGL), which causes the enhanced uptake of FFA across the cell membrane of hepatocytes through FA transporters, such as CD36, and the further re-esterification of FFA to form TG within hepatocytes [4,5,6]. At the hepatocyte level, the activation of SREBP-1c by LXRα in response to hyperinsulinemia also primarily induces FA and TG synthesis by up-regulating lipogenic genes, such as FA synthase and acetyl-CoA carboxylase (ACC), whereas the activation of SREBP-2 primarily promotes cholesterol biosynthesis by up-regulating cholesterogenic genes, such as HMGCR, a rate-limiting enzyme of the cholesterogenic process [4,6]. Consequently, an intermediate in lipogenesis, malonyl-CoA, can inhibit the rate-limiting enzyme involved in hepatic mitochondrial FA β-oxidation, CPT1, and LXR can also inhibit the rate-limiting enzyme in the clearance of circulating TG-rich lipoproteins, LPL, which may impair the oxidation of FA and clearance of circulating TG, respectively [6,7]. The abnormal lipid metabolism in the liver and adipose tissue results in hepatic steatosis, typically promoting the secretion of lipids, and hence, contributing to high circulating FFA levels, hypertriglyceridemia, and hypercholesterolemia [4,6]. Consistent with previous reports [20,21,26,27,28], our findings showed that HFD-fed animals presented the typical features of hepatic steatosis and hyperlipidemia, including high serum FFA, hypertriglyceridemia, and hypercholesterolemia, associated with the elevated expression of ATGL in adipose tissue, CD36, LXRα, SREBP-1c, SREBP-2, and HMGCR, as well as decreased CPT1A expression in the liver, which were overall reversed by RRBE treatments, especially at the middle and high doses. Interestingly, although a high-dose RRBE treatment did not reduce serum total-C in HFD-fed mice, it significantly attenuated LDL-C and enhanced HDL-C in the serum, as discussed in more detail below. The treatment of HFD-fed mice with RRBE also strongly increased the expression of the LPL gene in adipose tissue, although this gene was not affected by HFD. As observed in our previous animal study [11], RRBE could suppress the expression of the HSL and SREBP-1c genes, thereby reducing FFA and TG accumulation in white adipose tissue. Moreover, phenolic extract and hydrolyzed bound phenolics from rice bran, as well as other bioactive compounds such as proanthocyanidin, protocatechuic acid, oryzanol, vitamin E, and coenzyme Q10, modified the expression of key genes or proteins involved in hepatic lipid metabolism, such as CD36, LXRα, SREBP-1c, FA synthase, CPT1A, SREBP-2, and/or HMGCR, and led to the decrease in hepatosteatosis and hyperlipidemia in animal models with metabolic syndrome [14,29,32,33,34,35,36]. Our previous work showed the presence of phenolic compounds, including proanthocyanidins and anthocyanins, in RRBE [11]. Herein, we additionally observed the presence of protocatechuic acid, γ-oryzanol, vitamin E, and coenzyme Q10 in RRBE. Due to their lipid metabolism-regulating properties, these bioactive compounds present in RRBE may exhibit direct or synergistic ameliorating effects on the development of NAFLD and hyperlipidemia induced by HFD, which require further investigation. Collectively, this study reveals that the molecular mechanisms behind RRBE’s ability to alleviate hepatosteatosis and hyperlipidemia in the HFD mouse model are likely associated with the balanced expression of major genes involved in adipose tissue lipolysis (ATGL), hepatic FA uptake (CD36), hepatic FA and TG synthesis (LXRα and SREBP-1c), hepatic FA oxidation (CPT1A), hepatic cholesterol synthesis (SREBP-2 and HMGCR), and circulating TG degradation (LPL).

We further examined whether the treatment with RRBE resulted in elevated circulating HDL-C levels in the HFD-fed mouse model. It is well known that low levels of HDL, a tissue cholesterol-removing particle, are one of the components of atherogenic dyslipidemia in obesity and its associated diseases, including NAFLD [8]. The liver is not only an important organ responsible for the biogenesis of HDL components to form HDL particles, such as the major protein component ApoA-ǀ, but also for TG-rich HDL clearance in the blood through the activation of HL. An imbalance in this metabolism, with the increased clearance of circulating HDL and the impaired hepatic synthesis of HDL components, has been proposed as the mechanism for low HDL-C concentrations and functionality in systemic circulation [8]. As noted above, we found that serum total-C levels remained significantly higher in the obese group treated with RRBE at 1 g/kg/day than those in the control group and were similar to the untreated obese group. Remarkably, however, we demonstrated that despite the unchanged total-C levels, serum LDL-C levels were reduced, whereas the serum HDL-C levels were increased only in obese mice treated with a high-dose RRBE, which might thus reflect the increased levels of serum total-C in these mice. Also, RRBE at a higher dose was more effective at increasing HDL-C. This was accompanied by the down-regulation of HL and up-regulation of ApoA-ǀ in the liver. We, thus, propose that the mechanisms for the elevation of HDL-C levels after treatment with RRBE may be mediated by the down-regulation of the HL gene and up-regulation of the ApoA-ǀ gene in the liver, which attenuates HDL clearance and accelerates HDL ApoA-ǀ synthesis, respectively. These results are supported by previous studies showing that rice bran phenolic extract, protocatechuic acid, oryzanol, vitamin E, and coenzyme Q10 effectively raised blood HDL-C concentrations in NAFLD and dyslipidemic animals [32,35,36,37,38,39], and that vitamin E strongly increased the ApoA-ǀ levels [38], suggesting that these compounds contained in RRBE may exert positive effects on HDL homeostasis. However, the specific compounds responsible for these effects need to be further studied.

Finally, we examined whether RRBE inhibited the mechanisms involved in liver injury. In the advanced stage, the hepatic accumulation of toxic lipids could induce hepatic inflammation and injury [3,4,9], which represents high levels of aminotransferases, indicators of NASH [40]. Inflammation in NAFLD/NASH is regulated by the NF-κB signaling cascade, which can activate the transcription of genes encoding pro-inflammatory enzymes and mediators, such as iNOS, cytokines, and chemokines [4,9,30,41]. Pro-inflammatory mediators from stressed or dying liver cells, as well as immune cells, may promote the activation and infiltration of inflammatory cells into the liver, thereby damaging liver cells [4,42,43]. The activation of NF-κB by stress stimuli, such as ROS, also transactivates the expression of pro-oxidant enzymes (e.g., NOX), which, in turn, promote ROS production or oxidative stress and inflammation in the liver, thus favoring oxidative liver damage, such as lipoperoxidative damage, and eventually hepatocyte death [4]. Nicotinamide adenine dinucleotide phosphate oxidase can produce superoxide following the assembly of its cytosolic regulatory subunits (e.g., p47^phox^) with the membrane subunits (e.g., gp91^phox^) [9,44]. Moreover, hepatocyte injury and death from NALFD are also associated with apoptosis, which is involved in the imbalance of Bcl-2 family members, including enhanced levels of pro-apoptotic Bax molecules and/or reduced levels of anti-apoptotic Bcl-2 molecules [4,29]. In agreement with previous reports [21,29,30,31], our HFD-fed mouse model developed hepatosteatosis concomitant with elevated inflammatory markers (infiltrating inflammatory cells, NF-κB p65, iNOS, and NO), increased oxidative stress markers (ROS, MDA, and p47^phox^), and altered apoptotic markers (Bax and Bcl-2) in the liver, all of which might contribute to the hepatic damage as indicated by raised levels of AST and ALT. By contrast, these adverse effects were attenuated in HFD-fed mice to a certain extent by cotreatment with RRBE. In line with these data, previous in vivo studies have proved that RRBE, due to its high content of phenolics, could relieve HFD-induced white adipose tissue inflammation by attenuating inflammatory cell infiltration into this tissue and down-regulating the expression of NF-κB target genes, such as iNOS [11], and that RRBE also could inhibit paracetamol-induced hepatic necrosis by restoring the glutathione antioxidant system [45]. Furthermore, rice bran phenolics protected against ethanol-induced hepatic damage in mice through the suppression of the NF-κB inflammatory pathway, oxidative stress, and the Bax/Bcl-2 expression ratio [46,47]. Additional studies have revealed that bioactive compounds such as proanthocyanidin, protocatechuic acid, oryzanol, vitamin E, and coenzyme Q10 exhibit anti-inflammatory, antioxidant, anti-apoptotic, and/or anti-damaging effects in the livers of rodent NAFLD models [29,34,35,38,39], indicating that these bioactive compounds in RRBE may be responsible for the beneficial effects against liver damage in the present study. Consistent with its lipid-regulatory effects, our findings clearly suggest that RRBE could exert anti-inflammatory, antioxidant, and anti-apoptotic effects against HFD-induced hepatic injury by modulating the transcriptional expression of specific genes in the liver responsible for these effects, including the NF-κB target genes-iNOS, p47^phox^ NOX subunit, pro-apoptotic Bax, and anti-apoptotic Bcl-2, implying that RRBE may mitigate the transition to more severe stages of NAFLD. However, the effects of RRBE and its phytonutrients on other severe and end-stages of NAFLD, such as cirrhotic and liver failure stages, are unknown and require further studies.

## 5. Conclusions

This in vivo study gives new insight into the anti-hepatic steatosis and anti-dyslipidemic properties of RRBE. Using the HFD-induced NAFLD mice model, we demonstrate that RRBE can alleviate NAFLD and dyslipidemia by regulating the expression of lipid-regulatory genes, including FA/TG (↓ATGL, ↓CD36, ↑LPL, ↓LXRα, ↓SREBP-1c, and ↑CPT1A), cholesterol (↓SREBP-2 and HMGCR), and HDL (↓HL and ↑ApoA-ǀ) metabolic genes, in the liver and adipose tissues. Moreover, RRBE can also mitigate HFD-induced hepatic injury through anti-inflammatory (↓NF-κB and iNOS), antioxidant (↓p47^phox^), and anti-apoptotic (↓Bax and ↑Bcl-2) mechanisms. Therefore, RRBE should be considered an effective nutraceutical agent or food product for the management of NAFLD, dyslipidemia, and linked complications. Further analyses are needed to elucidate the effects of specific phytonutrients from RRBE and their possible novel mechanism of action against NAFLD and dyslipidemia.

## Figures and Tables

**Table 1:** Contents of bioactive constituents of RRBE.

Bioactive Constituents	Content
Protocatechuic acid (μg/g)	277.94 ± 7.69
γ-oryzanol (μg/g)	481.81 ± 18.80
Vitamin E (μg α-tocopherol/g)	3.18 ± 0.01
Coenzyme Q10 (μg/g)	9.81 ± 0.13

Values are means ± SD of triplicate analyses.

## Data Availability

The data presented in this study are available in the article.

## Supplementary Materials

The following supporting information can be downloaded at: https://www.mdpi.com/article/10.3390/nu15010246/s1, Table S1: TaqMan probe assay IDs and Figure S1: Effects of RRBE on initial body weight (A) and energy intake (B) in mice fed with experimental diet. Values are means ± SD; 6 mice/group. Statistically significant difference at *p* < 0.05 was analyzed using one-way ANOVA with Tukey’s test and is indicated by different letters above error bars. The L, LFD, and distilled water co-treated group; LR1, LFD, and high-dose (1 g/kg) RRBE co-treated group; H, HFD, and distilled water co-treated group; HR0.25, HFD, and low-dose (0.25 g/kg) RRBE co-treated group; HR0.5, HFD, and middle-dose (0.5 g/kg) RRBE co-treated group; HR1, HFD, and high-dose RRBE co-treated group.
