# Supplementary material for: Red Rice Bran Extract Alleviates High-Fat Diet-Induced Non-Alcoholic Fatty Liver Disease and Dyslipidemia in Mice
Source: Nutrients. 2023 Jan 3;15(1):246. doi: 10.3390/nu15010246 (PMC9824566; doi:10.3390/nu15010246)
Supplement: Supplementary file 1 [file nutrients-15-00246-s001.zip › nutrients-2137323-supplementary.pdf]

Table S1. TaqMan probe assay IDs.

Gene name	Assay IDs
ATGL	Mm00503040_m1
CD36	Mm00432403_m1
LPL	Mm00443451_m1
LXRα	Mm00434764_m1
SREBP-1c	Mm00550338_m1
CPT1A	Mm00550442_m1
SREBP-2	Mm01306292_m1
HMGCR	Mm01282499_m1
HL	Mm01171487_m1
ApoA-I	Mm00437569_m1
NF-κB p65	Mm00501346_m1
iNOS	Mm00440502_m1
p47phox	Mm00447921_m1
SOD1	Mm01344233_g1
CAT	Mm00437992_m1
Bax	Mm00432051_m1
Bcl-2	Mm00477631_m1
GAPDH	Mm99999915_g1

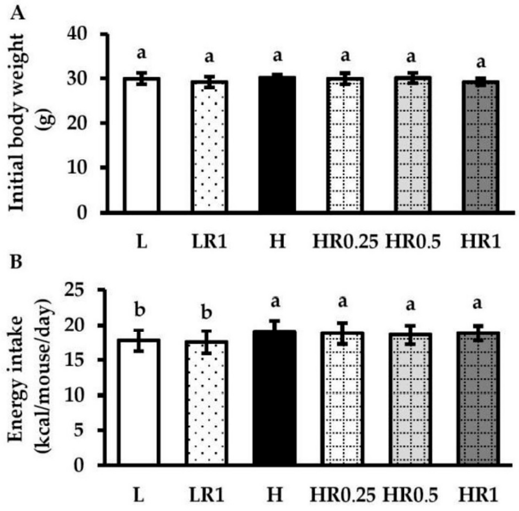

Figure S1. Effects of RRBE on initail body weight (A) and enery intake (B) in mice fed with experimental diet. Values are means ± SD; 6 mice/group. Statistically significant difference at $p < 0.05$ was analyzed using one-way ANOVA with Tukey's test and is indicated by different letters above error bars. L, LFD and distilled water co-treated group; LR1, LFD and high-dose (1 g/kg) RRBE co-treated group; H, HFD and distilled water co-treated group; HR0.25, HFD and low-dose (0.25 g/kg) RRBE co-treated group; HR0.5, HFD and middle-dose (0.5 g/kg) RRBE co-treated group; HR1, HFD and high-dose RRBE co-treated group.
